# Periodic protein‐restricted diets extend the lifespan of high‐fat diet‐induced *Drosophila* melanogaster males

**DOI:** 10.1111/acel.14327

**Published:** 2024-08-29

**Authors:** Ruohua Wang, Qiushuang Zhu, He Huang, Mengxia Yang, Xinyue Wang, Yuanjie Dong, Yuqiao Li, Yue Guan, Lei Zhong, Yucun Niu

**Affiliations:** ^1^ Department of Nutrition and Food Hygiene, College of Public Health, Key Laboratory of Precision Nutrition and Health, Ministry of Education Harbin Medical University Harbin Heilongjiang China; ^2^ Department of Breast Surgery Sixth Affiliated Hospital of Harbin Medical University Harbin Heilongjiang China

**Keywords:** dietary adjustments, fatty acid metabolism, high‐fat diet, lifespan, periodic protein‐restricted diet

## Abstract

Research has shown that sustained protein restriction can improve the effects of a high‐fat diet on health and extend lifespan. However, long‐term adherence to a protein‐restricted diet is challenging. Therefore, we used a fly model to investigate whether periodic protein restriction (PPR) could also mitigate the potential adverse effects of a high‐fat diet and extend healthy lifespan. Our study results showed that PPR reduced body weight, lipid levels, and oxidative stress induced by a high‐fat diet in flies and significantly extended the healthy lifespan of male flies. Lipid metabolism and transcriptome results revealed that the common differences between the PPR group and the control group and high‐fat group showed a significant decrease in palmitic acid in the PPR group; the enriched common differential pathways Toll and Imd were significantly inhibited in the PPR group. Further analysis indicated a significant positive correlation between palmitic acid levels and gene expression in the Toll and Imd pathways. This suggests that PPR effectively improves fruit fly lipid metabolism, reduces palmitic acid levels, and thereby suppresses the Toll and Imd pathways to extend the healthy lifespan of flies. Our study provides a theoretical basis for the long‐term effects of PPR on health and offers a new dietary adjustment option for maintaining health in the long term.

## Graphical abstract

Periodic protein‐restricted diet during the young and middle stages can reduce the body weight and lipid levels in high‐fat‐fed male flies and increase climbing ability, antioxidant capacity, and starvation resistance, thereby extending the flies' healthy lifespan. Further exploration into the mechanism revealed that the likely mechanism by which a periodic protein‐restricted diet extends a healthy lifespan is through reducing palmitic acid levels, thereby inhibiting the Toll and Imd antimicrobial immune inflammatory pathways and thus exerting longevity effects.

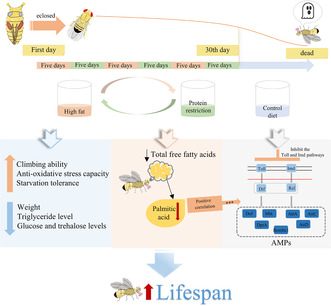

**Abbreviations**
AMP: antimicrobial peptide
GO: gene ontology
HFD: high‐fat diet group
KEGG: kyoto encyclopedia of genes and genomes
NAFLD: non‐alcoholic fatty liver disease
NC: normal diet group
OPLS‐DA: the orthogonal partial least squares discriminant analysis
PA: palmitic acid
PPR: periodic protein restriction
PR: protein‐restricted diet group

## 1 INTRODUCTION

The harmful effects of high‐fat diets on the body have been confirmed by numerous studies and are considered significant risk factors for conditions such as obesity, diabetes, and cardiovascular diseases (Her et al., 2020; Rasool et al., 2018). Continuous consumption of high‐fat diets, whether in experimental animals or humans, has been proven to lead to obesity and insulin resistance and increase inflammatory responses in the body, thus accelerating the aging process and reducing a healthy lifespan (Nayak & Mishra, 2021; Rasool et al., 2018). Adjusting dietary habits appropriately can effectively prevent and alleviate the development of metabolic diseases caused by high‐fat diets. Therefore, exploring the optimal dietary adjustment strategies to prevent and improve aging‐related metabolic diseases resulting from a high‐fat diet is a hot topic among nutritionists.

Current research on dietary adjustment strategies mainly falls into two categories: fasting and dietary restriction. Recent research indicates that fasting can reverse metabolic disturbances caused by a high‐fat diet, lowering glucose and triglyceride levels and improving insulin resistance (Barnosky et al., 2014; Salgado‐Canales et al., 2023). However, frequent and prolonged fasting may lead to malnutrition, exacerbating the body's burden and posing health risks. In contrast, dietary regimens that restrict energy or specific nutrients while meeting daily basic nutritional requirements have gained more popularity. The health effects of restrictive diets are closely linked to the levels of amino acids and proteins and their modulation of nutritional signaling pathways (Mirzaei et al., 2016). Therefore, restricting protein intake is considered a crucial dietary restriction strategy to prevent age‐related diseases and extend lifespans (Mirzaei et al., 2016).

Experimental studies on rodents have shown that restricting protein intake can help reduce obesity and insulin resistance caused by a high‐fat diet, possibly due to improvements in mitochondrial function (Branco et al., 2019). In addition, restricting specific amino acids such as branched‐chain amino acids, methionine, and cysteine can also improve high‐fat diet‐induced Non‐Alcoholic Fatty Liver Disease (NAFLD), thyroid dysfunction, and metabolic disorders by activating the AMPK alpha pathway and inhibiting mTORC1, thereby extending the health span of rodents (Lu et al., 2024; Wu et al., 2019; Yang et al., 2018). In studies using invertebrate Drosophila models, limiting yeast in the diet (the main source of protein for fruit flies) has been shown to reduce the activity of the nutrient‐sensing pathway TOR, thus increasing lifespan (Kapahi et al., 2004). However, both fasting and restricting protein diets require long‐term adherence to yield health benefits, posing challenges in terms of poor compliance among populations. If PPR could effectively prevent and ameliorate the health damages caused by a high‐fat diet, it would address the issue of low compliance associated with long‐term adherence.

Therefore, our study is focused on feeding young and middle‐aged male flies a high‐fat diet and implementing PPR interventions to observe if it can also effectively prevent the health damages caused by a high‐fat diet and lead to long‐term lifespan extension in the organism. Our research offers a novel dietary adjustment approach to mitigate the harm caused by high‐fat diets on health, providing reliable scientific evidence and recommendations for people's long‐term dietary plans.

## 2 MATERIALS AND METHODS

### 2.1 Drosophila rearing and dietary intervention plan

**FIGURE 1:**
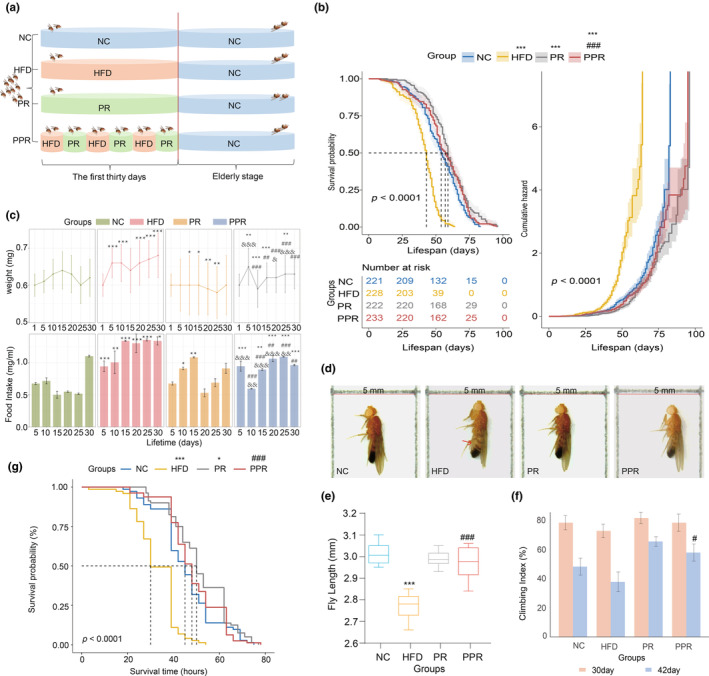
General biological characteristics and measurements of flies. (a) Experiment grouping flow chart. (b) Curve of survival. Survival analysis was performed using the log‐rank test. The curve on the left represents the survival curve of flies, indicating the survival status of flies over time. The curve on the right represents the cumulative hazard curve, showing the change in mortality cumulative risk of flies over time. (c) Effects of periodic restriction protein diet intervention on the body weight and food intake of Drosophila melanogaster. One‐way ANOVA and the Dunnett's test were used for analysis. (d) Drosophila body length morphology. The red arrow indicates the abdominal distension feature induced by a high‐fat diet, which is considered a sign of obesity. (e) The length of the fly. The differences in body length among groups were analyzed using a one‐way ANOVA and the Dunnett's test. (f) Measurement of climbing capacity. The differences in climbing index among groups were compared using the Chi‐square test. (g) Assessment of starvation resistance in Drosophila melanogaster at the elderly stage. Survival analysis under stravation was performed using the log‐rank test. The significance of differences between each experimental group and the control group is denoted by “*”, between the PPR group and the high‐fat group is denoted by “#,” and between the PPR group and the PR group is denoted by “&.” *, #, and & indicate *p* < 0.05; **, ##, and && indicate *p* < 0.01; ***, ###, and &&& indicate *p* < 0.001. NC denotes the control group; HFD, the high‐fat diet group; PR, the protein‐restricted diet group; PPR, the periodic protein‐restricted diet group.

The experiment used the *w* ^1118^ strain of Drosophila melanogaster purchased from Fangjing Biologicals in Qidong, Jiangsu Province, China. The flies were maintained in a constant‐temperature incubator at 25°C with 60% humidity and a 12‐h light–dark cycle. The breeding and control diets were based on and modified from the feed formula previously studied by Shi Dan et al. (2021) in our laboratory. In addition to the control diet formula, 10% lard was added as a high‐fat diet. The inclusion of yeast extract in our fly feed was a crucial protein source for the flies. The detailed feed formula can be found in Table S1. To prevent mating effects, virgin male flies of uniform size and good vitality within 4 h of eclosion from the breeding medium were divided into four groups for dietary intervention. The intervention was limited to the first 30 days. After 30 days, all groups of flies were fed the control diet. Notably, in the PPR group, we alternated between the high‐fat diet group (HFD) and the protein‐restricted diet group (PR) every 5 days within the initial 30 days, totaling three cycles (Figure 1a). All intervention groups of flies had their culture medium changed every 3 days, with a fly density of 20–25 flies per vial. Breeding vials were set up with approximately 40–50 flies, with an equal number of female and male flies for mating. After 3 days, the female parent flies were transferred to new breeding feed.

### 2.2 Survival experiment

In the survival experiment, we strictly controlled 20 flies per vial placed horizontally in a constant‐temperature incubator to prevent accidental contact with the culture medium. 300 flies were collected per group, and the number of naturally dead flies was counted at 2 PM every day, with feed changed every 3 days. The experiment concluded when all flies in each group had died, and survival curves were plotted for survival analysis.

### 2.3 Determination of body weight and assessment of consumed food volumes

To depict the dynamic changes in body weight during the dietary transition process, we selected groups of the same batch of flies to be weighed every 5 days during the initial 30‐day intervention phase. For each group, 100 fruit flies were anesthetized with ether, and 3–5 flies were weighed on a precision electronic balance (J500 Precision Electronic Balance) to obtain the average weight. The amount of food consumed by male flies in the various dietary interventions in this study was determined using the Brilliant Blue method, which we adopted and modified from the experimental protocol of Bross et al. (2005). (Details of the measurement procedure can be found in the supplementary materials).

### 2.4 Measurement and observation of body length and morphology

The flies were anesthetized with ether for 42 days, and their body lengths were measured using a vernier caliper (from the head to the tip of the wing) with 30 fruit flies in each group. Subsequently, the anesthetized flies were placed under an electron microscope (Binyun lens 500×) for morphological observation and photography, with each group experiment repeated three times. The body length of flies was measured using a 5 mm grid paper placed flat under a microscope as a scale. (Figure S1).

### 2.5 Climbing ability assessment

The 30‐ and 42‐day‐old flies were transferred to culture tubes marked with an 8‐cm horizontal line. The tubes were gently shaken to make the flies fall to the bottom of the tubes, and then the flies' normal physiological behavior of climbing up along the inner wall of the tube was utilized. The number of flies reaching the horizontal line on the tube within 12 s was counted. This number was recorded five times for each tube, and the average was calculated (Manjila & Hasan, 2018).

Climbing index=number of fliesonthe horizontal linetotal number×100%

### 2.6 Starvation tolerance test

According to the experimental protocol of Cannell et al. (2016), we placed each group of Drosophila at day 42 in water agar placed horizontally in a constant‐temperature incubator and observed and counted the number of deaths every 6 h. Each vial contained 20 flies, and there were three vials per group. The experiment was terminated when all the flies had died, and survival curves were plotted for survival analysis.

### 2.7 Biochemical index detection

We measured six biochemical indicators of fruit flies in each group at 30 and 42 days, namely trehalose, glucose, triglycerides, catalase, superoxide dismutase, and glutathione. Except for glucose, which was measured using a fully automatic biochemical analyzer (ROCHE Diagnostics), the other indicators were measured using assay kits (manufacturer: Nanjing Jiancheng and Solarbio). Each experiment was repeated three times per group. (Details of the measurement procedure can be found in the supplementary materials).

### 2.8 Lipid droplet Nile red fluorescence staining

Our experimental method adopted and improved the approach of Nayak et al. (2021), in which groups of flies at 30 and 42 days were subjected to fat body and intestinal dissection (mainly extracting the midgut) and fixed overnight in 4% paraformaldehyde at 4°C. The samples were washed with PBS three times for 5 min each. Staining of the samples was done with a working concentration of 0.5 μg/mL Nile Red, followed by a 1‐h incubation. The excess dye was then removed, and all samples were washed with PBS for 5 min. The stained fat bodies and intestines were flattened and placed on slide loadings, followed by the addition of 50 μL anti‐fluorescence quenching mounting medium containing DAPI (Biosharp). A coverslip was gently placed over the sample, ensuring complete light avoidance throughout. Subsequently, the samples were examined and imaged under a laser confocal microscope. ImageJ 5.0 software was utilized for the measurement of lipid droplet diameter and area.

### 2.9 Fatty acid profile test

Utilizing the TRACE 1310 gas chromatograph equipped with a helium TSQ 8000 Evo mass spectrometer (Thermo Finnigan, Austin, TX, USA), we performed GC/MS–MS analysis to measure the levels of free fatty acids (FFA) in Drosophila melanogaster (120 ± 10 mg, age = 42 days). The method for detecting the metabolism of fly fatty acid profiles and the sample weight were determined based on previous research in our laboratory and preliminary experiments conducted. (Shi, Han et al. 2021). (Details of the measurement procedure can be found in the supplementary materials).

### 2.10 Transcriptome sequencing

RNA sequencing technology was employed to analyze 42‐day‐old male fly samples to explore the gene changes and mechanisms that may lead to the extension of lifespan in PPR. Initially, total RNA was extracted from the samples using TRIzol, and its quality, purity, and integrity were assessed. Subsequently, mRNA containing polyA was selectively screened and fragmented using oligo (dT) magnetic beads and a magnesium ion cleavage reagent. The fragmented RNA was then used to synthesize cDNA and undergo double‐strand synthesis, followed by end repair and A‐tailing, as well as selection and purification. Next, a size‐specific library of fragments, approximately 300 bp ± 50 bp, was generated through PCR amplification. Finally, paired‐end sequencing using the PE150 mode on the Illumina platform was performed. Post‐sequencing, data filtering was conducted to obtain high‐quality, clean data. Subsequently, these data were compared to the reference genome of the project species for gene expression quantification, differential gene analysis, enrichment analysis, and more. The raw sequencing data was uploaded to the NCBI database (Accession number: PRJNA1094602).

### 2.11 Quantitative real‐time PCR

According to the manufacturer's instructions, total RNA was isolated from whole Drosophila flies (*n* = 20, three replicates per group) using TRIzol reagent (Ambion, TX, USA). The RNA was reverse transcribed into cDNA using the High Capacity cDNA Reverse Transcription Kit (Applied Biosystems, CA, USA). Real‐time PCR was performed using SYBR Green PCR Master Mix and the Roche480 Real‐time PCR system (Applied Biosystems, CA, USA). The Drosophila gene primers were purchased from Sangon Biotech Company (Jiangsu, China). According to the references, Rp49 had been identified as a housekeeping gene in flies (Kabil, Kabil et al., 2011). The detailed sequence of the primer can be found in Table 1, and the relative gene expression levels were calculated using the Ct value method.

**TABLE 1:** Real‐time quantification of PCR primers.

Gene	Forward primer sequence	Reverse primer sequence
Rp49	TGCTAAGCTGTCGCACAAATGG	TGCGCTTGTTCGATCCGTAAC
AttC	TACCCAGCCACTGATCTCCA	TTAGGTCCAATCGGGCATCG
Def	CAGCCAGTTTCCGATGTGGA	GAGTAGGTCGCATGTGGCTC
AttD	TCGGTGATGATCTTGCCAATGC	TGCATGACCATTGGCGTTGAC
Rel	ACACCGCCAAGAAGTACATATTCG	GCCTCACGCTCTGTCTCCTG
Mtk	GCTACATCAGTGCTGGCAGAG	GGTCTTGGTTGGTTAGGATTGAAGG
Bombc1	AAGTGCCTGATTCTGTCCTTTGC	ATACTCCGCCGATAATCACATTTCC
DptA	CAGTCCAGGGTCACCAGAAGG	CAAGTGCTGTCCATATCCTCCATTC
AttA	TTCCTTGACGCACAGCAACTTC	GTAGCACGATTGGGCGATGAC
Imd	GGGATCTTGGCATGTCGGAA	AGCTTCGAATCCACTGGAGC
Tl	CGGACATCGGAGATGTGGAG	TTACCGATATTGCCGACGGG
Dif	CTTTTAAGACGCCGCGCTAC	CACGGCGATTGTGTTTGGTT

### 2.12 Integrated analysis of Core gene selection, fatty acid metabolism, and transcriptome

Screened out the differentially expressed genes for the comparison groups of the normal diet group (NC) versus PPR and HFD versus PPR, based on the criteria fold Change (FC) ≥2 and *p* < 0.05. Selected the intersection genes of these two gene sets as the differential gene set for core gene screening. Analyzed the interaction of differential genes using the STRING website (https://cn.string‐db.org/) to obtain a gene network diagram with interacting genes. Subsequently, used cytoscape software to select the top 7 differentially expressed genes as the core genes identified through screening. We conducted inter‐omics correlation analysis of the 61 common differentially expressed genes between PPR, NC, and HFD and free fatty acids on the MetWare Metabolomics Cloud Platform (https://cloud.metware.cn/#/home). The analysis method employed Pearson correlation analysis. For molecular pairs with a correlation coefficient greater than 0.4 and a *p*‐value less than 0.05, they were considered significantly correlated. Subsequently, we used cytoscape software to construct a correlation network of the differential molecules.

### 2.13 Statistical analysis

The overall survival analysis for lifespan and starvation resistance data was conducted using the log‐rank test. The categorical data on the climbing ability of flies were analyzed using the chi‐squared test. For other continuous variables, if not specified otherwise, the standard approach involved using one‐way ANOVA followed by Dunnett's post hoc test for statistical analysis. The data analysis was primarily performed using R (version 4.2.0) and SPSS (IBM SPSS Statistics 25). A *p*‐value less than 0.05 was considered statistically significant. The interpretation of results is as follows: *p* ≥ 0.05 indicates no statistical significance; *p* < 0.05 is denoted by “*”, indicating significance; *p* < 0.01 is denoted by “**”; and *p* < 0.001 is denoted by “***”.

## 3 RESULTS

### 3.1 PPR significantly extends the lifespan of male flies

Current research indicates that a high‐fat, high‐energy diet will decrease the lifespan of fruit flies, while dietary restriction (typically achieved by limiting total energy or yeast content in fruit fly studies) is widely believed to extend lifespan (Kapahi et al., 2004; Nayak & Mishra, 2021). Our study reveals that, compared to the NC group, the HFD group of flies exhibited a significant 22.6% reduction in average lifespan, whereas the PR group (with a 50% reduction in yeast content compared to the control diet) showed a significant extension of 13.2% in average lifespan, consistent with existing literature. (Figure 1b; Table S2) The PPR intervention in flies led to a significant 5.7% increase in average lifespan compared to the control group. (Figure 1b; Table S2) The cumulative mortality risk curve also demonstrates that over time, the cumulative risk of death in the PPR group is lower than that in the HFD and NC groups, corresponding to the survival curve. (Figure 1b) This indicates that PPR intervention can effectively reverse the shortened lifespan caused by a high‐fat diet and significantly extend the lifespan of male fruit flies, with no significant difference in lifespan compared to the PR group (*p* > 0.05).

### 3.2 The dynamic changes in body weight and food intake of flies during the intervention stage (1–30 days post‐eclosion)

The most direct manifestation of restricted dieting is weight loss. To observe the impact of PPR on the weight of flies, we measured the weight of the same batch of flies at each diet change point (every 5 days) and found that the most significant weight decrease occurred only during the first cycle of PPR. Subsequent cycles showed a stabilization in weight, as depicted in Figure 1c. Overall, PPR can significantly reduce the impact of high‐fat feeding on fly weight gain (compared to the HFD group). Studies have shown that mice subjected to restricted diet feeding and then returned to normal dieting exhibit an increase in food intake, which may be a key factor contributing to weight rebound (Rosenbaum et al., 2019). Therefore, we dynamically measured the food intake of flies at 5‐day intervals. The results indicated that, apart from a significant increase in food intake after the first feeding of a PR diet, the food intake of the flies in the following two cycles exhibited a similar trend to weight changes (with no significant fluctuations), as shown in Figure 1c. Overall, the food intake of flies on a PPR diet was significantly lower compared to the HFD group (*p* < 0.05). It is worth noting that there was no difference in food intake between the PR group and the control group, indicating that the impact of PR on the healthy lifespan of flies is not due to energy restriction but rather protein restriction.

### 3.3 The performance of flies in body length morphology, climbing ability, and starvation tolerance under different dietary interventions

#### 3.3.1 Body length morphology

The body length and morphology of flies can, to some extent, reflect their development and health status (Nayak & Mishra, 2021). Therefore, we measured the body length of flies in each group at 42 days. The results showed that the body length of the PPR group was not significantly different from the NC and PR groups but significantly increased compared to the HFD group (Figure 1d,e). Current literature indicates that flies fed a high‐fat diet have relatively shorter body length and abdominal swelling (a sign of fruit fly obesity; Nayak & Mishra, 2021; as shown in Figure 1d, red arrows). Flies in our HFD group also exhibited the same characteristics. However, the PPR group flies had no abdominal swelling (Figure 1d). Therefore, it can be seen that PPR can effectively prevent fly obesity induced by a high‐fat diet, thereby exhibiting a good physical condition.

#### 3.3.2 Climbing ability

The important indicator for measuring the physical activity status of flies is the assessment of climbing ability, which indirectly reflects the aging status of flies (Tuo et al., 2023). Therefore, we measured the climbing abilities of flies in each group at early intervention completion (day 30 of adulthood) and in the elderly stage (day 42 of adulthood), respectively. The results showed that at day 30, the climbing ability of the PPR group of flies was relatively higher compared to the HFD group (*p* > 0.05). As age increased, the climbing ability of the HFD group of flies decreased the fastest, while the decline rate of climbing ability in the PPR group was comparable to the PR group. By day 42, the climbing ability of the PPR group of flies was significantly higher than that of the HFD group (*p* < 0.05) (Figure 1f). Thus, it can be seen that undergoing PPR in the young and midlife stages can significantly enhance the climbing ability of flies and delay the aging process of the body.

#### 3.3.3 Starvation tolerance

As the aging process progresses, the starvation tolerance of flies weakens, and the stronger starvation tolerance of flies indicates a stronger ability to mobilize body fat, the younger the fly's body (Baldal et al., 2006). We observed that the average starvation duration (hours) of flies in the PPR group fed only agar at 42 days was extended by 10.9% compared to the NC group and by 48.5% compared to the HFD group. Analysis of the fly starvation survival data showed that there was no significant difference in starvation tolerance between the PPR group and the NC and PR groups, while there was a significant increase in starvation tolerance compared to the HFD group (*p* < 0.05) (Figure 1g). Based on current research on starvation resistance and aging, we have further confirmed the beneficial effects of PPR on the healthy lifespan of flies fed a high‐fat diet.

### 3.4 Detection of sugar and lipid metabolism indicators in drosophila, fluorescent staining of lipid droplets, and assessment of oxidative stress‐related markers

#### 3.4.1 Sugar metabolism indicators

**FIGURE 2:**
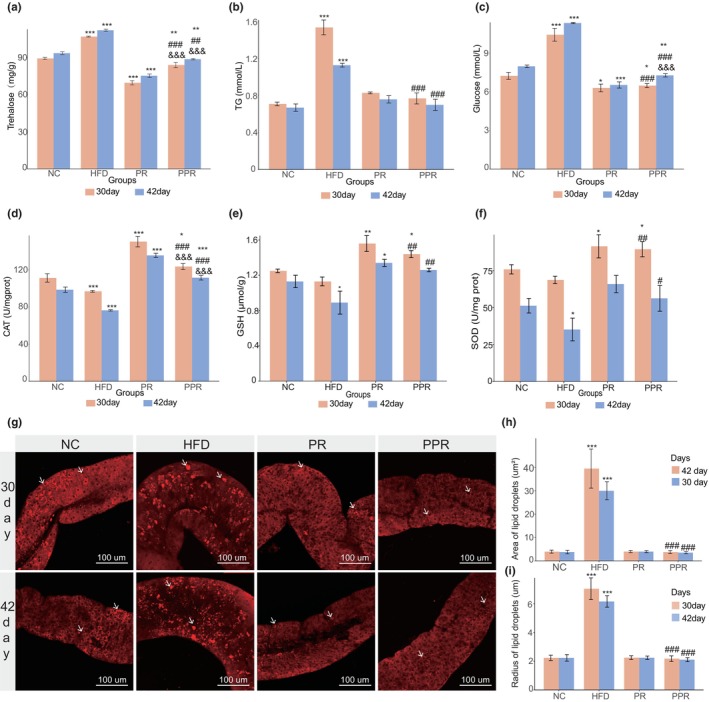
Determination of Drosophila triglyceride and oxidative stress markers, and nile red staining of intestinal lipid droplets. (a) The levels of trehalose, (b) triglyceride, (c) glucose, (d) catalase, (e) glutathione, and (f) superoxide dismutase. One‐way ANOVA and the Dunnett's test were used for analysis. (g) The fluorescence of lipid droplets in the intestine of Drosophila melanogaster. The white arrows point to lipid droplets in various groups of intestines. (h) Measurement of intestinal lipid droplet area and (i) radius. One‐way ANOVA and the Dunnett's test were used for analysis. The significance of differences between each experimental group and the control group is denoted by “*”, between the PPR group and the high‐fat group is denoted by “#,” and between the PPR group and the PR group is denoted by “&.” *, #, and & indicate *p* < 0.05; **, ##, and && indicate *p* < 0.01; ***, ###, and &&& indicate *p* < 0.001. NC denotes the control group; HFD, the high‐fat diet group; PR, the protein‐restricted diet group; PPR, the periodic protein‐restricted diet group.

In fly research, a high‐fat diet increases the sugar content in fly bodies, leading to the induction of fly insulin resistance and metabolic disorders such as diabetes (Nayak & Mishra, 2021; Yan et al., 2023). Our study found that regardless of whether it was for 30 days or 42 days, the sugar indicators (trehalose and glucose) in the PPR group flies were significantly lower than those in the NC and HFD groups, as shown in Figure 2a,c. This indirectly indicates that the PPR diet can effectively improve the increased circulating sugar levels caused by a high‐fat diet, thereby preventing the occurrence of sugar metabolic disorders in flies.

#### 3.4.2 Lipid metabolism indicators and lipid droplet fluorescence staining

Research has shown that a high‐fat diet can lead to an increase in triglyceride levels in Drosophila, thereby disrupting sugar metabolism and causing an elevation in the organism's sugar levels (Nayak & Mishra, 2021). As one of the main lipid storage organs in Drosophila, the intestine exhibits a significant increase in lipid droplets after being subjected to high‐fat feeding (Nayak & Mishra, 2021). For the sake of comparability of lipid droplets, we uniformly selected lipid droplets in the midgut R2 region for comparison (the anatomical structures of the intestinal R2 region can be seen in Figure S2). The research findings indicate that a high‐fat diet feeding regimen leads to an increase in triglyceride levels and an enlargement of lipid droplets in the Drosophila intestine, which is consistent with current research results (Figure 2b,g–i). In contrast, the triglyceride content, intestinal lipid droplet area, and diameter in the PPR group did not differ significantly from the NC and PR groups but were significantly reduced compared to the HFD group (Figure 2b,g–i). The smaller size of lipid droplets in the fat body tissue of flies in the PPR group compared to the HFD group (Figure S3a–c) further strongly indicates that PPR can effectively reduce high‐fat diet‐induced fat accumulation, thereby preventing obesity.

#### 3.4.3 Oxidative stress‐related indicators

As the aging process progresses, the level of oxidative stress in the body also increases, and the body's antioxidant capacity can indirectly reflect its health and aging status (Edrey & Salmon, 2014; Taylor et al., 2022). Therefore, we examined the activities of SOD enzyme, CAT enzyme, and the content of reduced glutathione in 30‐day and 42‐day flies to observe the antioxidant capacity of flies in different intervention groups. The results showed that at the end of the 30‐day intervention, the three oxidative stress indicators in the PPR group flies were significantly higher than those in the NC and HFD groups. Even 12 days after the cessation of periodic dietary protein restriction intervention, the antioxidant capacity of the PPR group flies remained significantly higher than that of the HFD group (*p* < 0.05) and slightly higher than that of the NC group (*p* > 0.05). (Figure 2d–f) It can be seen that PPR can not only reverse the oxidative damage caused by a high‐fat diet but also enhance the antioxidant capacity of flies, thereby effectively delaying the aging process.

### 3.5 Changes in fatty acid metabolism profile

**FIGURE 3:**
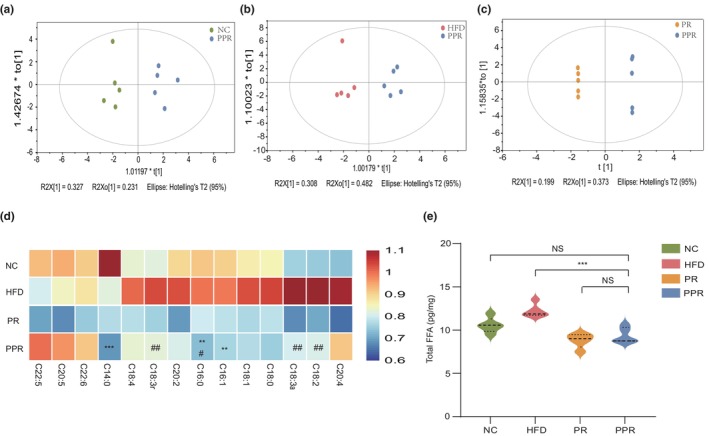
Effects of different dietary interventions on the fatty acid metabolism profile of elderly flies. (a–c) OPLS‐DA analyses of the PPR group compared to the control groups NC, HFD, and PR. (d) A heat map of the levels of 14 types of free fatty acids in different dietary intervention groups. (e) Comparison of total free fatty acid content among different dietary intervention groups. ANOVA and the Dunnett's test were used for analysis. “NS” is interpreted as “no statistically significant difference.” Significance levels between the PPR group and the control group were denoted by “*”, between the PPR group and the high‐fat group by “#,” and between the PPR group and the PR group by “&.” *, #, and & *p* < 0.05; **, ##, and && *p* < 0.01; ***, ###, and &&& *p* < 0.001. NC, control group; HFD, high‐fat diet group; PR, protein‐restricted diet; PPR, periodic protein‐restricted diet.

In order to further observe the impact of PPR intervention on the fat metabolism of flies, we measured the profiles of free fatty acid metabolism in the various groups of flies at 42 days. The Orthogonal Partial Least Squares Discriminant Analysis (OPLS‐DA) analysis revealed significant differences between the PPR group and the NC, HFD, and PR groups, with relatively small differences within each group. (Figure 3a–c). Further permutation tests confirmed that none of the three models exhibited overfitting (Q2 <0), indicating their capability to effectively discriminate between samples and generalize to new data sets, thus demonstrating a certain level of reliability. (Figure S4) After calculating the total fatty acid content of the four groups of flies, we found that the total fatty acid content of the PPR flies was significantly lower than that of the HFD group, while the difference compared to the NC and PR groups was not significant (Figure 3e), consistent with the results of triglyceride and intestinal lipid droplet staining. Summarizing the content of 14 free fatty acids in each group of flies and presenting it in a heatmap, we observed that the content of most fatty acids in the PPR group (except for C22:5, C20:5, and C22:6) was lower compared to the NC and HFD groups of flies (Figure 3d). From the 14 free fatty acids, we identified one common free fatty acid, palmitic acid (C16:0), that showed significant differences (*p* < 0.05, VIP value >1) in comparisons between PPR versus NC and PPR versus HFD groups (Figure 3d). We believe that PPR likely acts by improving fatty acid metabolism, significantly reducing palmitic acid (PA) levels, and thereby impacting the lifespan of flies.

### 3.6 Transcriptome analysis of elderly male flies in different intervention groups

#### 3.6.1 Principal component analysis and differential gene expression

**FIGURE 4:**
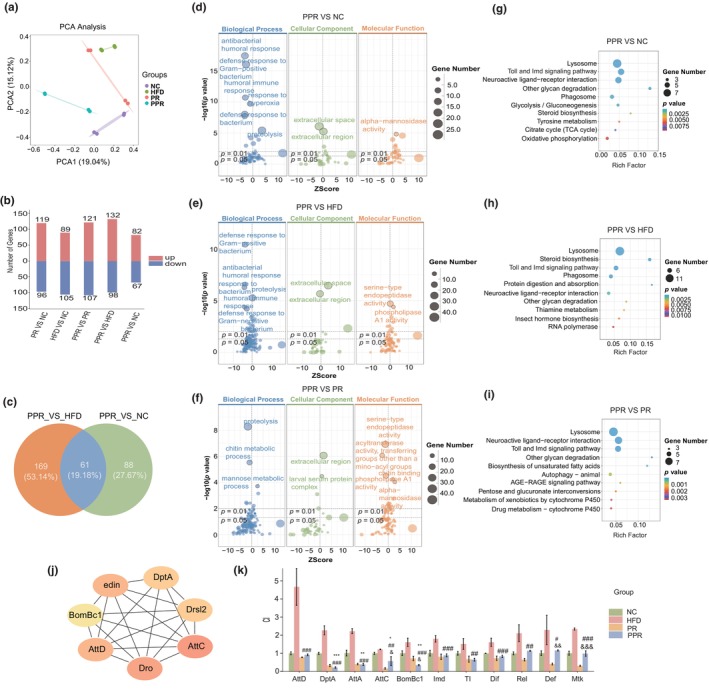
Transcriptome analysis of Drosophila, core gene selection, and PCR validation. (a) PCA analysis of four groups of flies. (b) The number of differentially expressed genes in flies between each comparative group. (c) Venn diagram of differentially expressed genes between the PPR group and the NC and HFD comparison groups. The intersection portion represents the common differential genes. (d–f) GO enrichment analysis. (g–i) KEGG enrichment analysis. A Z‐score less than 0 indicates pathway downregulation, while a Z‐score greater than 0 indicates pathway upregulation. (j) Core Gene Screening. The deeper the red color, the higher the gene is ranked. (k) PCR analysis. One‐way ANOVA and the Dunnett's test were used for analysis. Significance levels between the PPR group and the control group were denoted by “*”, between the PPR group and the high‐fat group by “#,” and between the PPR group and the PR group by “&.” *, #, and & *p* < 0.05; **, ##, and && *p* < 0.01; ***, ###, and &&& *p* < 0.001. NC, control group; HFD, high‐fat diet group; PR, protein‐restricted diet; PPR, periodic protein‐restricted diet.

The PCA analysis revealed well‐defined clustering within groups and distinct differences between groups (Figure 4a). The within‐group correlation analysis showed Pearson correlation coefficients above 0.97 for each group (Figure S5a), indicating the similarity and reproducibility of samples within each group, thus validating the reliability of the transcriptomic results. By comparing the expression levels of differentially expressed genes (*p* < 0.05 and FC ≥2) between PPR versus NC, HFD, and PR (Figure 4b and Figure S5b–d), we observed 61 common differentially expressed genes in the intersection between PPR versus NC and PPR versus HFD (Figure 4c).

#### 3.6.2 PPR group differentially expressed genes are significantly enriched in the fly immune antimicrobial pathway

After conducting Gene Ontology (GO) and Kyoto Encyclopedia of Genes and Genomes (KEGG) analyses to understand the differences in significantly expressed genes related to biological functions between PPR and NC, HFD, and PR comparisons, we found that the GO analysis for PPR versus NC and HFD comparisons primarily converged on immune and antimicrobial‐related pathways, all of which were significantly downregulated (Figure 4d,e). The KEGG analysis identified the top three pathways enriched in the Toll and Imd pathways (fly immune and inflammatory pathways), which were consistent with the GO enrichment in immune antimicrobial pathways (Figure 4g,h). Although the KEGG analysis for PPR versus PR also significantly enriched the Toll and Imd pathways among the top three pathways, the GO analysis did not show a significant upregulation or downregulation of immune antimicrobial‐related pathways (Figure 4f,i). Therefore, we speculate that the extended lifespan in flies due to PPR intervention compared to the NC and HFD groups is likely associated with the Toll and Imd pathways.

#### 3.6.3 Core gene screening

To explore the potential mechanisms by which PPR extends lifespan, we identified the top seven core differential genes (DptA, Drsl2, AttC, Dro, AttD, BomBc1, and edin) among the 61 common differential genes in the PPR, NC, and HFD groups (Figure 4j). Interestingly, these seven core genes are all associated with the anti‐bacterial immune response in flies. Apart from edin, the remaining six core genes are key genes in the Toll and Imd pathways, further supporting our hypothesis that PPR extends lifespan by downregulating the Toll and Imd pathways.

#### 3.6.4 The main genes on the Toll and Imd pathways show reduced expression in the PPR group

To validate the transcriptomic analysis results and our hypothesis that PPR inhibits the Toll and Imd pathways to extend the lifespan of flies, we conducted PCR verification on all key genes in the Toll and Imd pathways. The results showed that downstream antimicrobial peptide (AMP) genes (AttD, DptA, AttA, AttC, Def, Mtk, and BomBc1) in the PPR were significantly lower than those in the NC and HFD groups. While the inflammatory immune genes Dif and Rel did not differ significantly from the NC group, they were significantly lower than in the HFD group. Furthermore, the upstream genes Tl and Imd in the pathway were also significantly lower in the HFD group (Figure 4k). Overall, most genes in the Toll and Imd pathways of flies in the PPR group were significantly downregulated compared to the NC and HFD groups, with less distinct differences from the PR group. The PCR validation confirmed the results of the transcriptomic sequencing, further supporting the notion that inhibiting the Toll and Imd pathways may have beneficial effects on the lifespan and health of flies.

### 3.7 Integrated analysis of free fatty acid metabolism and transcriptome

**FIGURE 5:**
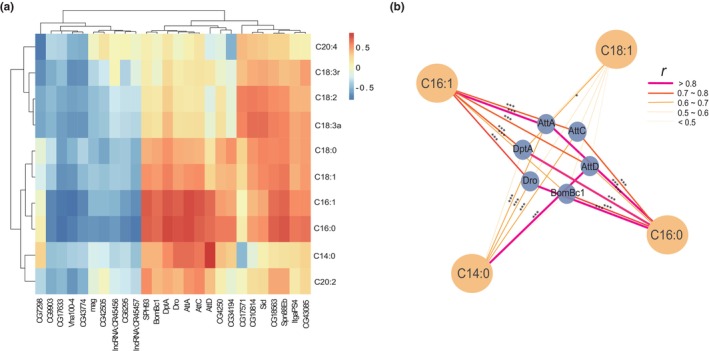
Integrative analysis of transcriptome and fatty acid metabolism profiles. (a) Correlation Heatmap. A heatmap showing the correlation between differentially expressed genes shared by PPR, NC, and HFD comparison groups and 14 types of free fatty acids. Red indicates a positive correlation, while blue indicates a negative correlation. (b) Relevance Network Diagram. We displayed the differentially expressed genes and free fatty acids with a correlation coefficient greater than 0.4. *A p*‐value less than 0.05 indicates statistical significance. *p < 0.05; **p < 0.01; ***p < 0.001. NC, control group; HFD, high‐fat diet group; PR, protein‐restricted diet; PPR, periodic protein‐restricted diet.

To explore the potential mechanisms underlying the extension of PPR lifespan, we conducted a correlation analysis between PPR and 61 common differentially expressed genes related to NC and HFD, as well as free fatty acids. Interestingly, we found a significant positive correlation between 6 AMP genes (DptA, Dro, AttA, AttC, BomBc1, and AttD) in the Drosophila Toll and Imd pathways and 14 types of free fatty acids (Figure 5a). Further network analysis revealed that C16:1, C14:0, and C16:0 were significantly positively correlated with most of these AMP genes, with correlation coefficients greater than 0.6 for C16:0 with AMP genes (Figure 5b). Notably, C16:0 (palmitic acid) is also a common differential fatty acid in PPR versus NC and HFD. Therefore, we further speculate that PPR may prolong a healthy lifespan by reducing levels of palmitic acid, thereby suppressing Drosophila immune inflammation levels.

## 4 DISCUSSION

A high‐fat diet has been shown to increase body fat storage, leading to the occurrence of metabolic diseases such as obesity and ultimately reducing healthy lifespan in various biological models and human studies (Baenas & Wagner, 2022; Boutagy et al., 2015; Ren et al., 2022). Our research has similarly confirmed that a high‐fat diet can result in adverse health effects in Drosophila melanogaster, including increased fat accumulation, reduced stress tolerance, elevated sugar and lipid levels, and a shortened lifespan. We discovered that subjecting high‐fat‐fed male flies in their young and midlife stages to three cycles of 5‐day periods of restricted protein diet reduces body weight, has no abdominal obesity features, significantly enhances antioxidant stress response, and increases starvation resistance, demonstrating that PPR can effectively prevent obesity and reverse the adverse health effects of a high‐fat diet. Even after discontinuation of this intervention, the health benefits persist and significantly extend the healthy lifespan of flies. We believe that such a dietary adjustment strategy, which does not require long‐term adherence, does not pose any potential health risks of malnutrition, and ensures long‐lasting health benefits, is more easily accepted and implemented.

No matter whether it is dietary restriction or fasting, the most immediate effect after a period of time is the reduction in body weight. Studies on periodic dietary restriction in rats have shown that the first cycle of dietary restriction can reduce the body weight of rats by 20%. With an increase in cycles, more time is required to achieve another 20% weight reduction. However, overall, periodic cycling in rats gradually reduces body weight, leading to a sustained decrease in obesity levels and improved glucose tolerance (Rosenbaum et al., 2019). Our fly research indicates that the initial 5‐day continuous restriction of protein intake significantly reduces body weight. Subsequent cycles show a lower decrease in weight and improvements in sugar and lipid levels, consistent with the findings in rat studies. Although HFD flies exhibit increased fat storage, their starvation resistance is far inferior to that of PPR flies. What are the reasons for this contradiction? Katewa et al. (2012) utilized radiolabeling techniques to demonstrate that restricting protein intake enhances triglyceride synthesis and breakdown rates in PR‐fed flies. Thus, we speculated that the underlying reason for this contradiction may lie in the fact that PPR flies possess a higher capacity for fat metabolism, thus better adapting to starvation conditions. The accelerated triglyceride metabolism in PR‐fed flies is likely mediated by the IRE1/XBP1 endoplasmic reticulum stress signaling module, which is essential for longevity extension in PR‐fed flies. It is further hypothesized that interventions promoting longevity in PPR‐fed flies are closely associated with lipid metabolism (Luis et al., 2016). Imbalance in fat synthesis and breakdown leads to the continuous accumulation of fat in the organism. The triacylglycerol content in fly bodies, as well as the size of lipid droplets in storage organs (such as the fat body and intestine of flies), can visually reflect the accumulation of fat (Nayak & Mishra, 2021). For male flies fed a high‐fat diet, we found that the phenomenon of fat accumulation induced by a high‐fat diet can be completely prevented and reversed after three cycles of periodic protein dietary restriction.

Fatty acid metabolic profiling results showed a significant decrease in total fatty acid content in PPR flies compared to the high‐fat group, further demonstrating that PPR can prevent and reverse the adverse health effects of fat accumulation induced by a high‐fat diet. Additionally, the common differential fatty acid, PA, was significantly lower in the PPR group compared to the control group and high‐fat group, suggesting a close relationship between PA and the extension of lifespan in flies. Obesity induced by a high‐fat diet increases PA levels, which can upregulate the expression of intracellular TLR2 and TLR4, generate reactive oxygen species (ROS), increase NF‐κB activity, induce the expression and activity of Toll‐like receptors (TLR), and release inflammatory factors (Dasu & Jialal, 2011; Qiu et al., 2022). The NF‐κB signaling pathway is an important inflammatory pathway, and PA has been shown to increase host inflammatory responses (Hidalgo‐Lanussa et al., 2018; Jin et al., 2013; Korbecki & Bajdak‐Rusinek, 2019; Sergi et al., 2021). Aging induces the expression of inflammation‐related genes in the livers of mice (Acosta‐Rodríguez et al., 2022). In previous studies on significantly extended lifespans of rats and fruit flies by isoenergetic high‐fat diets, our combined analysis of proteomics and metabolomics also revealed the association of palmitic acid with organismal inflammation and lifespan, confirming that isoenergetic high‐fat diets can significantly reduce palmitic acid levels, upregulate PPRC1 through PPARG, ultimately extending the healthy lifespan of rats and fruit flies, and improve oxidative stress and inflammatory status (Shi, Han et al. 2021). PPARG is an important regulator in fatty acid metabolism, involved in regulating inflammatory responses in adipose tissue (Hamaguchi & Sakaguchi, 2012). NF‐κB, as an ancient signaling pathway, has been found in insects and vertebrates. Research identifies the NF‐κB system, a major regulator of innate immunity, as the culprit behind age‐promoting inflammatory responses. As aging progresses, the immune system also ages, and innate immunity seems to be activated, triggering characteristic pro‐inflammatory responses (Kanigur Sultuybek et al., 2019; Micolucci et al., 2023; Rahman et al., 2022).

In the fly organism, there are two unique NF‐κB signaling pathways, namely the Toll signaling pathway and the Imd signaling pathway, which are utilized to combat different pathogens. The Toll signaling pathway primarily combats most gram‐positive bacterial and fungal infections, while the Imd signaling pathway mainly targets most gram‐negative bacterial infections (Valanne et al., 2011). These two pathways activate different NF‐κB transcription factors through transmembrane receptor‐mediated signaling cascades, regulating the expression of a series of target genes, including the production of AMPs. The Toll signaling pathway activates the transcription factors Dorsal and Dif by degrading the IκB‐like inhibitor cactus (Valanne et al., 2011), whereas the Imd signaling pathway activates the transcription factor relish by cleaving its internal inhibitory domain (Myllymäki et al., 2014). Studies have shown a causal relationship between high levels of AMPs and neurodegenerative diseases in flies; as flies age, NF‐κB‐dependent AMP gene expression increases, accompanied by progressive neurodegeneration and decreased motor abilities (Kounatidis et al., 2017). To further demonstrate the close causal relationship between the Toll and Imd NF‐κB pathways and the extension of fly lifespan, reducing the normal levels of NF‐κB in healthy fly brains resulted in an extended lifespan and improved activity in old age (Kounatidis et al., 2017). In vitro experiments have also shown that the combined inhibition of the Toll/NF‐κB pathway in insulin‐producing cells (IPCs) and neuroblasts increases fly lifespan, and even inhibiting Toll/NF‐κB in IPCs alone is sufficient to enhance survival under various lethal stresses, contributing to the extension of healthy fly lifespan (Khor & Cai, 2020). Our study similarly found that inhibiting the two important NF‐κB pathways (Toll and Imd) involved in the innate immune response of flies can improve organism activity and extend a healthy lifespan.

Current research has shown that a decrease in organismal PA levels inhibits the inflammatory response in the aging process, particularly by suppressing NF‐κB, thereby indirectly extending the healthspan of the organism (Alnahdi et al., 2019; Binker‐Cosen et al., 2017; Mangali et al., 2019). In our prior studies on the mechanisms underlying the lifespan extension in rats and fruit flies induced by an isocaloric high‐fat diet, we have demonstrated that fruit flies fed on the PA+ isocaloric high‐fat diet exhibit elevated oxidative stress and inflammation levels, with an upregulation of dorsal in the Toll pathway. Further overexpression of the PPRC1 gene was found to decrease the expression levels of the dorsal gene in PA‐fed fruit flies, thus ameliorating oxidative stress and inflammation levels in the organism (Shi et al., 2021). Therefore, we speculate that the extension of lifespan in male flies through PPR intervention is also likely achieved by lowering PA levels to inhibit the Toll and Imd pathways. Therefore, we conducted a combined analysis of fatty acid metabolism and the transcriptome. Genes on the NF‐κB pathways of both flies showed a positive correlation with free fatty acids, and the top six genes most correlated with the four types of fatty acids were all related to the Toll and Imd pathways. This indicates the relevance of fatty acid levels to the fly's antimicrobial immune pathways. Of note, among the four free fatty acids screened, PA exhibited the highest correlation with the six genes, further confirming the close association between PA and lifespan.

In conclusion, PPR intervention can effectively reduce fat accumulation in high‐fat diet‐fed male flies, enhance organism stress tolerance (to starvation and oxidative stress), and increase climbing activity levels, thereby extending a healthy lifespan. The potential mechanism underlying lifespan extension is that PPR intervention lowers the level of PA in fly organisms, thereby inhibiting the expression levels of genes related to the Toll and Imd pathways, ultimately prolonging the healthy lifespan of flies. Our research findings provide a novel theoretical basis for individuals seeking to maintain health through a healthy dietary intervention strategy. We look forward to the PPR dietary adjustment becoming an option for people who want to maintain long‐term health.

## 5 CHALLENGES AND PROSPECTS IN RESEARCH

Our research provides a novel dietary adjustment strategy to effectively prevent the harmful effects of poor eating habits and improve overall health. However, the future promotion of PPR diets faces the following challenges: (1) While PPR every 5 days has been shown to extend lifespan and is safe in flies, further research is needed to determine the optimal duration and frequency of protein restriction in other animal models and human populations. (2) The application and effects of PPR diets across different age groups and physiological conditions require ongoing research and refinement.

## AUTHOR CONTRIBUTIONS

RW designed the study. RW and QZ performed the experiments, analyzed the data, and wrote the manuscript. YL, YD, XW, and YG performed the experiments. HH and MY analyzed the data. YN and LZ critically reviewed the manuscript. All authors read and approved the final manuscript.

## CONFLICT OF INTEREST STATEMENT

The authors declare no conflict of interest.

## Supporting information


Data S1.

## Data Availability

The data that support the findings of this study are available from the corresponding author upon reasonable request.
