# Supplementary material for: Periodic protein‐restricted diets extend the lifespan of high‐fat diet‐induced Drosophila melanogaster males
Source: Aging Cell. 2024 Aug 29;23(12):e14327. doi: 10.1111/acel.14327 (PMC11634745; doi:10.1111/acel.14327)
Supplement: Supplementary file 1 — Data S1. [file ACEL-23-e14327-s001.docx]

**Supplementary Material**

1. **Materials and Methods Supplement**
   1. **Drosophila** **feed formula and preparation process**

**Drosophila** diet formulation and detailed ratio are shown in **Table S1**. The process of preparing 1000 ml of fly culture medium: 1. Pour 1200 ml of sterilized distilled water under high pressure (add 200 ml more distilled water than the intended culture medium to account for water evaporation during preparation). 2. Heat half of the added distilled water, and another half dissolve 50g of cornmeal without clumps. 3. Once the distilled water reaches boiling point, add 5.5g of agar, stir thoroughly until transparent, and then add the clump-free cornmeal suspension. Stir for 10 minutes, then add accurately measured sucrose and glucose before pouring into a beaker to reach the final volume. It is important to note that after adding the boiling cornmeal to the HFD medium, 100g of lard should be added along with Tween 80 (1 mL/100 mL) and mixed thoroughly. 4. Let it cool naturally to around 60 degrees Fahrenheit, add yeast, stir well, then introduce propionic acid (3 ml/1000 ml medium) and 20% Tegosept (15 ml/1000 ml medium). Once the feed is thoroughly mixed, quickly inject 5 ml into sterilized culture tubes using a sterile syringe (3-5 ml per tube).

Table S1. Recipes for experimental analysis of fruit flies (w/v)

| Component (g) | NC | HFD | PR |
| --- | --- | --- | --- |
| Agar | 5.5 | 5.5 | 5.5 |
| Glucose | 55 | 55 | 55 |
| Corn meal | 50 | 50 | 50 |
| Sucrose | 30 | 30 | 30 |
| Yeast | 18 | 18 | 9 |
| 20%Tegosept | 15 | 15 | 15 |
| Propionic acid | 3 | 3 | 3 |
| Lard | 0 | 100 | 0 |
| Water | 1000 ml | 1000 ml | 1000 ml |
| Total energy | 655.31 kcal | 1555.31 kcal | 554.97 kcal |

NC, normal control diet. HFD, high-fat diet. PR, restricted protein diet.

- 1. **Food intake measurement**

To determine the feeding status at each five-day interval during the dietary intervention period in the first 30 days, feeding measurements were taken at six time points: Day 5, Day 10, Day 15, Day 20, Day 25, and Day 30. Prior to the feeding measurements, flies in each group were starved for 2 hours in agarose with no food. Subsequently, the flies were transferred to vials containing 200 μl of 0.2% FD&C no. 1 blue dye-infused filter paper corresponding to each group (to avoid dye contamination on the flies, filter papers were prepared two hours in advance and dried thoroughly in an incubator). After feeding for 30 minutes, the flies were anesthetized with ether and then washed with PBS to remove any surface dye contamination. Three replicate samples of 20 flies each per group were then collected, flash-frozen in liquid nitrogen, and stored at -80°C for later analysis.

Once all fly feeding samples were collected, the flies were transferred to homogenization tubes and ground thoroughly in a homogenizer with 1 ml of PBS added. The homogenate was centrifuged, filtered through a 0.1 μm membrane, and 200 μl of the supernatant was pipetted into a 96-well plate with three replicate wells per sample. The absorbance was measured at a wavelength of 625 nm using a spectrophotometer. Absorbance measurements of different concentrations of FD&C no. 1 blue dye were used to construct a standard curve. The feeding amount of the flies was calculated using the standard curve, and the feeding amounts of the flies in each group were determined.

- 1. **Detection of Glycolipids and Oxidative Stress Biomarkers**

**1.3.1 Trehalose**

Before processing the samples, weigh the weights (g) of 20 flies on a balance and record the numbers (with three replicates for each group). Then, place them in pre-cooled centrifuge tubes in an icebox and add 1 ml of extraction solution. Homogenize thoroughly in a homogenizer three times, let it stand at room temperature for 45 minutes, and shake 3-5 times. Centrifuge at 8000g at room temperature for 10 minutes and keep for further analysis.

Prepare blank tubes (3 replicates), standard tubes (3 replicates), and test tubes (3 replicates for each sample). Follow the instructions of the alginate determination kit (anthrone colorimetric method 50T/48 samples), add various reagents, mix well, cover tightly, heat at 95°C for 10 minutes in a water bath, cool under running water, vortex mix, and transfer 200 microliters from each tube to a 96-well plate (ensuring no air bubbles). Measure the absorbance at 620nm for each well using a microplate reader. Calculate the content of alginate using the formula provided in the kit manual for tissue samples.

**1.3.2 Glucose**

Twenty flies are taken as one sample (with three replicates) and placed in a centrifuge tube, each labeled accordingly. 1ml of pre-chilled phosphate-buffered saline (PBS) is added to the tube, followed by thorough homogenization three times. The sample is then centrifuged at 8000g at room temperature for 10 minutes. After centrifugation, 200μl of the supernatant is transferred to a sample cup and placed on a fully automated biochemical analyzer for glucose measurement.

**1.3.3** **Triglyceride**

Twenty flies were placed in each centrifuge tube for each sample (with three replicates per sample). Subsequently, 1 ml of pre-chilled PBS was added to the tubes, followed by thorough homogenization three times. The suspension was then centrifuged at 2500 rpm for 10 minutes, and the supernatant was collected for further analysis.

For the experiment, blank wells (three replicates), standard wells (three replicates), and test wells (three replicates per sample) were set up. Following the instructions of the triglyceride assay kit (GPO-PAP enzymatic method, single reagent type, microplate method), various reagents and the supernatant of each sample were added to a 96-well plate. The plate was shaken well and then incubated at 37°C for 10 minutes. Subsequently, the absorbance values at a wavelength of 510 nm for each well were measured using a microplate reader. After determining the protein concentration of each sample using the BCA assay kit (Thermo Fisher Scientific), the triglyceride content was calculated using the formula provided in the kit's instructions.

**1.3.4 Catalase (CAT)**

Twenty flies are placed in each sample tube (with three replicates per sample) and numbered. 1ml of pre-cooled physiological saline is added, followed by thorough homogenization three times. The homogenate is then centrifuged at 2500 rpm for 10 minutes, and the supernatant is collected for testing.

Control wells (three replicates) and test wells (three replicates) are set up in a 96-well plate. Following the instructions from the hydrogen peroxide test kit (ammonium molybdate method), various reagents and the supernatant of each sample are added to the plate. After shaking the plate for homogenization, the absorbance values at a wavelength of 405nm for each well are measured using a microplate reader. Protein concentrations of the samples are determined using the BCA assay kit, and the catalase (CAT) activity values are calculated using the formula provided in the kit's instructions based on the tissue CAT activity.

**1.3.5** **Superoxide dismutase (SOD)**

Twenty flies were placed in each centrifuge tube (with three replicates per sample) and numbered. 1ml of pre-chilled SOD extraction solution was added, followed by thorough homogenization three times. The homogenate was then centrifuged at 8000g at 4°C for 10 minutes, and the supernatant was collected and stored in a cooler for testing.

Three replicate wells were set up for the test holes, control holes, blank tube 1, and blank hole 2. Following the instructions of the Superoxide Dismutase Activity Assay Kit (WST-1 method), various reagents and the supernatant samples were added to a 96-well plate. After shaking and incubating at 37°C for 30 minutes, the absorbance values at a wavelength of 450nm for each well were measured using a microplate reader. The protein concentration of each sample was determined using the BCA assay kit, and the SOD activity values were calculated based on the formula provided in the kit's manual.

**1.3.6** **Glutathione (GSH)**

Before processing the samples, weigh the weight of 20 flies on a balance and record the numbers (with three replicates for each group). Then, place them in pre-cooled homogenizer tubes in an icebox and add 1 ml of pre-cooled GSH extraction solution. Thoroughly homogenize the mixture three times. Subsequently, centrifuge the suspension at 3500 rpm for 10 minutes and collect the supernatant into the icebox for further analysis.

Set up blank wells (3 replicates), standard wells (3 replicates), and test wells (3 replicates). Following the operating instructions of the Microplate Glutathione Assay Kit (96T microplate method), add various reagents and the supernatant to the 96-well plate, gently shake the plate to mix, and let it stand for 5 minutes. Measure the absorbance values at a wavelength of 405nm for each well using a microplate reader. Calculate the GSH content based on the formula provided in the kit manual for tissue samples.

- 1. **The detailed steps for Drosophila fatty acid determination**

**1.4.1 Sample pretreatment**

1) Weighed approximately 120 mg of Drosophila on a balance and transferred them to a homogenizer tube, then added 1 ml of PBS into the homogenizer. Homogenized thoroughly three times.

2) Transferred the homogenized suspension to a 15-ml EP tube (labeled with a number on the tube) and added 1.5 ml of pre-prepared isopropanol:n-hexane (3:2) to it. Vortexed for 1 minute, let it stand for 2 hours, centrifuged at 3500 rpm for 10 minutes, separated into three layers, extracted the upper organic phase, and transferred it to a new 10 ml glass EP tube.

3) Added another 1.5 ml of isopropanol:n-hexane (3:2) to the remaining tissue residue and aqueous phase, vortexed for 1 minute, let it stand for 2 hours, centrifuged at 3500 rpm for 10 minutes, and extracted the upper organic phase again.

4) Combined the two organic phases and evaporated under nitrogen gas.

**1.4.2 Transesterification Process**

1) After drying, 0.2 ml of internal standard solution (200 μg/ml, C17:0, C17:0 methyl ester) was added into a 10 ml glass EP tube. Subsequently, 2 ml of the prepared 0.4 M potassium hydroxide methanol solution was added, and the mixture was stirred for 1 minute before being left at room temperature for 10 minutes.

2) A certain amount of anhydrous sodium sulfate and 2 ml of n-hexane were added into the glass EP tube. After stirring for 1 minute, the tube was centrifuged at 3500 rpm for 5 minutes and evaporated under nitrogen gas.

3) 0.1 ml of n-hexane was used to dissolve the sample, and after vortexing for 1 minute, 1 μl was taken and injected into the mass spectrometer for analysis.

1. **Results Supplement**

**2.1 Supplement Survival Data Table S2**

Table S2. Effects of periodic restriction of protein intake during the middle-aged stage on the lifespan of Drosophila

| Lifespan | NC | HFD | PR | PPR |
| --- | --- | --- | --- | --- |
| Average lifespan (days) | 53 | 41 ^***^ | 60 ^***^ | 56 ^*; ###; &^ |
| Rate of life extension/ loss (-) | - | -22.6% | +13.2% | +5.7% |
| Median lifespan (days) | 54 | 43 | 59 | 57 |
| Rate of life extension/ loss (-) | - | -20.4% | +9.3% | +5.6% |
| Maximum lifespan (days) | 77 | 56 ^***^ | 87 ^***^ | 81 ^*; ###; &&^ |
| Rate of life extension/ loss (-) | - | -27.3% | 13% | 5­­.2% |

^1^ The average lifespan of the top 10% longest-lived flies is the maximum lifespan. One-way ANOVA and the least significant difference test were used for analysis. The significance of differences between each experimental group and the control group is denoted by "*", between the PPR group and the high-fat group is denoted by "#," and between the PPR group and the PR group is denoted by "&." ^*, #,^ and ^&^ *P < 0.05*; ^**, ##,^ and ^&&^ *P < 0.01*; ***, ^###,^ and ^&&&^ *P < 0.001*. NC, control group; HFD, high-fat diet group; PR, protein-restricted diet; PPR, periodic protein-restricted diet.

**2.2**


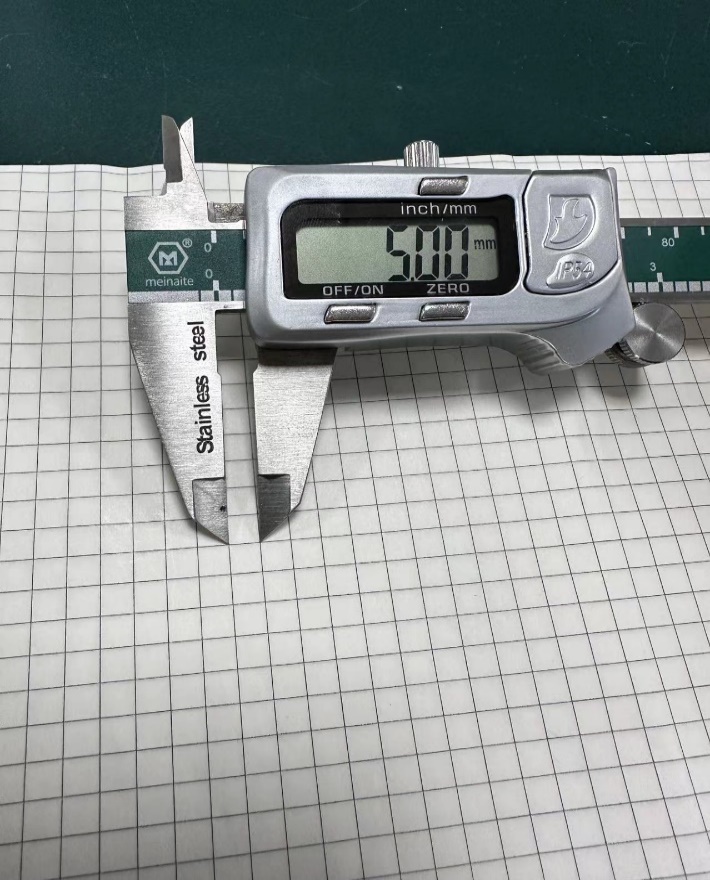


Figure S1 The grid side length.

**
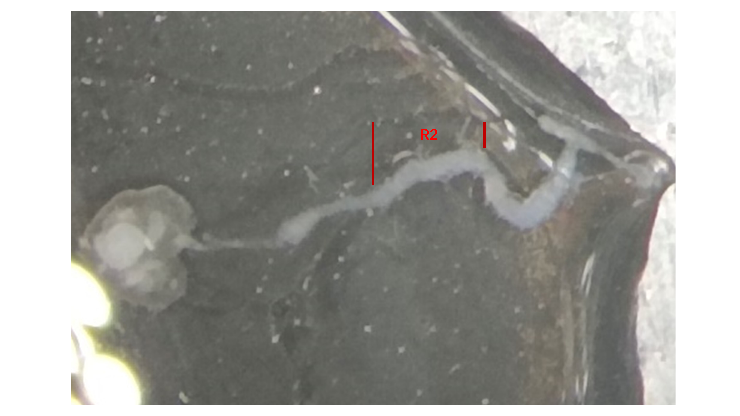
**

Figure S2 The anatomical structures of the intestinal R2 region

- 1. **Fluorescent Staining of Fatbody Lipid Droplets**

**
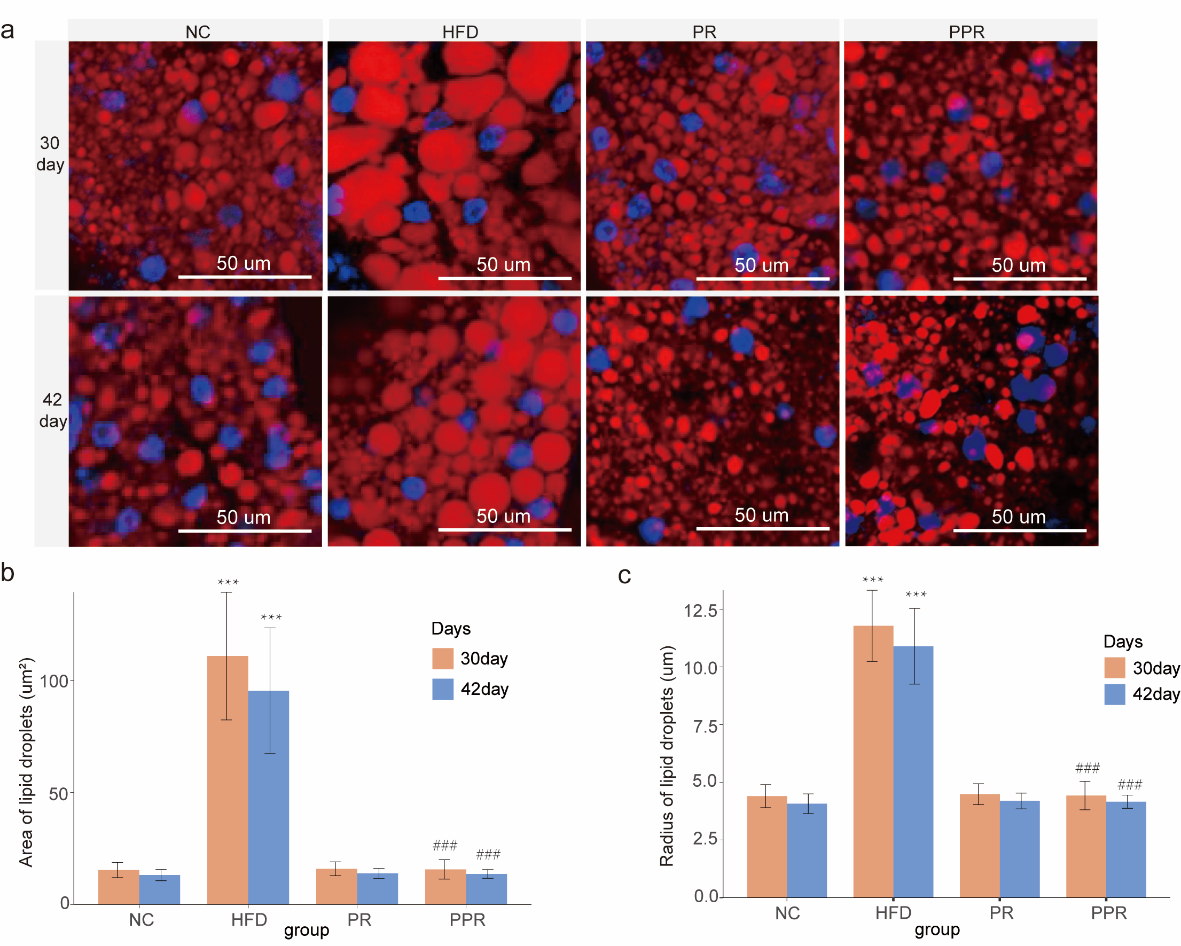
**

Figure S3: The fluorescence of lipid droplets in the fat body of Drosophila melanogaster. (b) Measurement of fat body lipid droplet area and (c) Radius. One-way ANOVA and the Dunnett test were used for analysis. The significance of differences between each experimental group and the control group is denoted by "*", and between the PPR group and the high-fat group is denoted by "#". "*", "#", and "&" indicate P < 0.05; "**", "##", and "&&" indicate P < 0.01; "***", "###", and "&&&" indicate P < 0.001. NC, control group; HFD, high-fat diet group; PR, protein-restricted diet; PPR, periodic protein-restricted diet.

- 1. **Permutation tests**

**
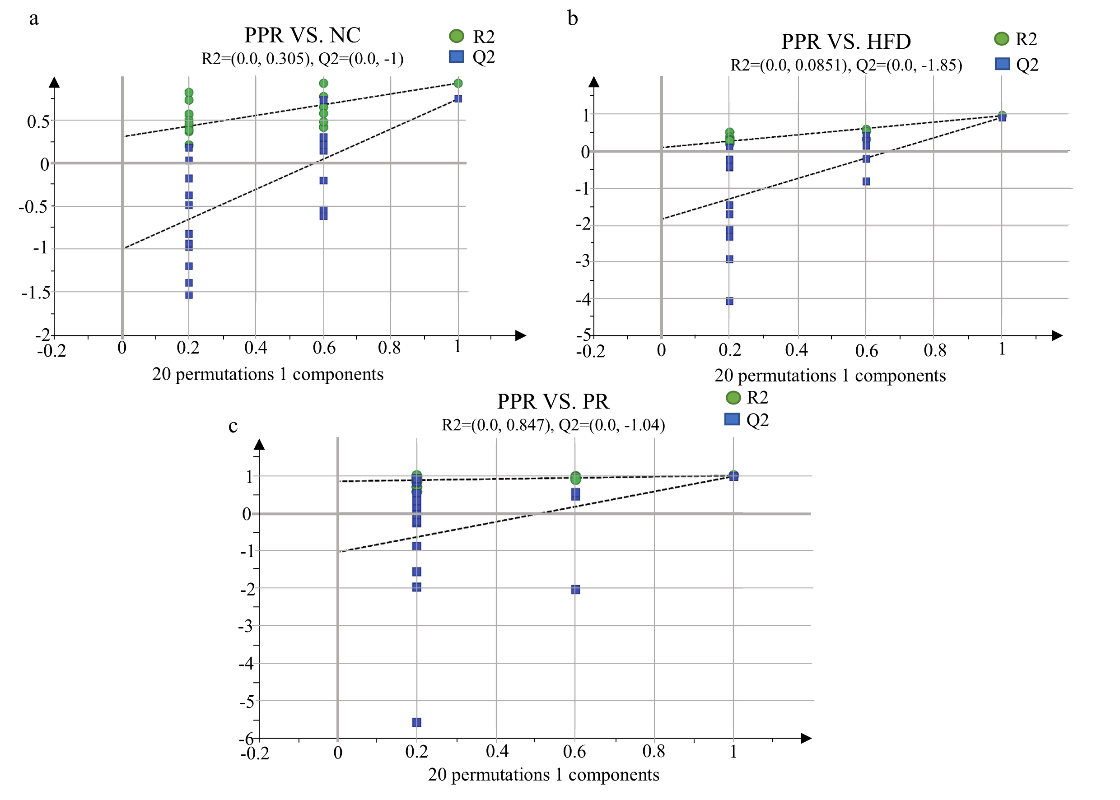
Figure S4** If the intercept of the Q2 regression line on the vertical axis is less than 0, it demonstrates the reliability of the model and indicates the absence of overfitting. (a) PPR vs. NC (b) PPR vs. HFD (c) PPR vs. PR

**2.6 Correlation analysis within groups and volcano plot of differentially expressed genes.**

**
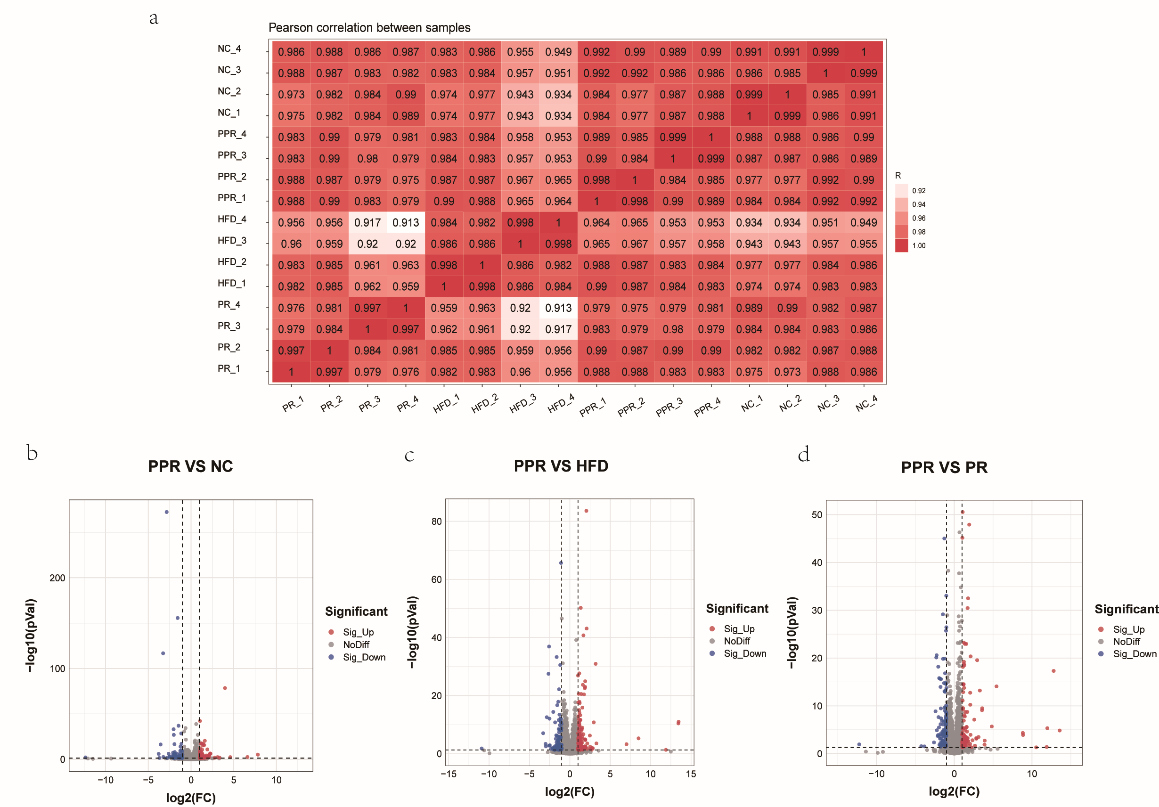
**

**Figure S5** Correlation coefficient heatmap and volcano plots of differentially expressed genes. (a) Correlation coefficient heatmap. (b, c and d) volcano plots. In a volcano plot, the horizontal axis represents the degree of gene expression difference, while the vertical axis represents statistical significance.
